# False memories and biased judgments for physical touch: the role of misinformation on eyewitness reports

**DOI:** 10.3389/fpsyg.2025.1724728

**Published:** 2026-02-20

**Authors:** Fabiana Battista, Tiziana Lanciano, Pasquale Zappimpulso, Federico Puleo, Ivan Mangiulli, Antonietta Curci

**Affiliations:** Department of Education, Psychology and Communication, University of Bari Aldo Moro, Bari, Italy

**Keywords:** misinformation, false memories, correct memories, interrogative suggestibility, touch

## Abstract

**Introduction:**

Memory is a reconstructive process susceptible to external influences. The misinformation effect, extensively studied in eyewitness testimony, refers to the distortion of post-event information upon memory recall. However, limited research has examined how misinformation influences memory along with evaluation of an event involving physical touch.

**Methods:**

Participants of the present study (*N* = 184) watched a video depicting a professor-student interaction including a physical touch (male professor/female student vs. female professor/male student), followed by a free and cued recall, and evaluative ratings. After 1 day, participants received either neutral or misleading post-event information (i.e., the professor working on teaching materials vs. the professor being under investigation for sexual harassment of students) and completed a second recall and rating session. Measures of interrogative suggestibility, working memory, and executive functioning were also assessed.

**Results:**

Results showed that misinformation significantly increased memory errors and influenced evaluative judgments over time, particularly in the male professor/ female student condition. Participants exposed to misinformation judged the professor’s behavior as more inappropriate and severe, and recommended a harsher punishment. A high level of individual’s suggestibility was associated with lower perceived intentionality, independent of exposure to misinformation.

**Discussion:**

These findings support the effect of misinformation on memory and judgments, highlighting critical implications for legal contexts.

## Introduction

The concept of memory as a transformative system, rather than a mere repository of information, challenges the storage room assumption where memories are considered fixed entities preserved intact over time (Curci, 2022; Frenda et al., 2011; Loftus, 1997; Roediger et al., 2014; Schacter, 1999). Indeed, memory is far from being a static record. It can diverge significantly from actual events during both the encoding and retrieval phases. Factors such as attention, the meaning attributed to an experience, and pre-existing world knowledge, organized as “schemas” and “scripts,” can influence the perception of an event, ultimately shaping how it is experienced and subsequently remembered (Alba and Hasher, 1983; Bransford and Johnson, 1972; Pickrell et al., 2016; Schacter et al., 2007). Furthermore, new information can be integrated into a memory subsequently, or it can be adapted to conform to one’s cognitive schemas, with external suggestions potentially leading to modifications as well. Consequently, when individuals recall an event, they may end up remembering details that are either partially inaccurate or completely false (Arnold and Lindsay, 2002; Loftus and Pickrell, 1995; Nash et al., 2015; Roediger and McDermott, 1995).

A considerable body of research has demonstrated that false memories can be easily induced using various experimental approaches, ranging from strictly controlled laboratory methods to more ecologically valid procedures that simulate clinical and legal scenarios (Otgaar et al., 2018). People can generate two main types of false memories: spontaneous false memories, which arise without any external prompting, and suggestion-induced false memories, which are shaped by external suggestions and post-event information (Brainerd et al., 2008; Loftus, 2005; Otgaar et al., 2023; Mazzoni et al., 1999). Moreover, some studies have also demonstrated that external information can influence evaluative judgments about an event (Kassin et al., 2003; Huntley and Costanzo, 2003; Melinder et al., 2020). Both these lines of research provide profound implications for real-world scenarios, particularly in the context of eyewitness testimony.

### External information and memory effects in legal contexts

One of the most used paradigms for studying false memories due to post-event information is the misinformation one (e.g., Loftus, 1975; Loftus et al., 1978; Battista et al., 2024). This approach follows a three-phase process. Initially, participants are exposed to stimuli, such as videos or photographs, depicting a crime or a car accident. In the second phase, they receive post-event information, often through a narrative or suggestive questioning, in which some details are altered (for example in Loftus et al., 1978, a stop sign might be incorrectly reported as a yield sign). When later questioned about the original event, individuals exposed to this misinformation frequently recall the altered details as if they were part of the original scene. A key finding from this research is that a notable proportion of participants incorporates the misinformation into their original recollections. The robustness of the misinformation effect is well-established, with numerous studies replicating these findings (see Loftus, 2005, for a comprehensive review).

A prominent theoretical explanation for this effect is provided by the Source Monitoring Framework (SMF; Johnson et al., 1993). This framework posits that memories are stored together with source information (e.g., when, where, and how the information was encoded). During retrieval, individuals engage in source monitoring, a cognitive process used to determine the origin of their memories. When misinformation is introduced after the event, individuals may experience source misattribution, confusing externally provided information (e.g., from an interviewer or misleading report) with details derived from their own experience. Because this external information can resemble the real one, people may erroneously attribute misleading details to the original event, leading to memory distortions (Mitchell and Johnson, 2000).

Alternatively, other scholars have proposed that the misinformation effect occurs because of a less accessibility of the original memory traces as compared with the external one, the so-called coexistence hypothesis (Christiaansen and Ochalek, 1983). The main assumption of this hypothesis is that both the original memory and the misleading post-event information continue to exist in memory simultaneously. However, since the misleading information is more recent than the encoded one, it is much more easily retrievable. Hence, although the memory traces of the original event still exist, people are more inclined to retrieve the misleading information. Also, the Fuzzy-Trace Theory (Brainerd and Reyna, 1998) has been used to justify the misinformation effect (e.g., Brassil et al., 2024). According to the theory, when we encode an episodic memory, we create two parallel representations of the same experience. The verbatim trace preserves the exact perceptual details of what occurred, while the gist trace captures the essential meaning or general interpretation of those details. Gist traces are more open to distortions than verbatim memories (Brainerd and Reyna, 1998; Reyna et al., 2016), therefore the misinformation effect is due to alteration of gist traces rather than verbatim.

Research has also shown that discussing the event with one or more co-witnesses is another prominent source of memory contamination, demonstrating the so-called memory conformity effect (Gabbert et al., 2003; Mojtahedi et al., 2017; Tousignant et al., 1986; Wright et al., 2000). Some studies on memory conformity found that exposure to a co-witness statement (i.e., written statement) could even lead to blame on an innocent person for perpetrating a crime (Mojtahedi et al., 2018a; Mojtahedi et al., 2018b; Thorley, 2015). This phenomenon, termed as blame conformity (Thorley and Rushton-Woods, 2013), occurs when people alter their memory such that they blame the same person accused by the co-witness (Mojtahedi et al., 2018a; Mojtahedi et al., 2018b; Thorley, 2015). Mojtahedi et al. (2020) investigated whether individual characteristics, such as age and gender, could influence blame conformity. The authors detected no gender and age-differences in blame attribution and conformity, such that men and women reported similar blame attribution when exposed to co-witness misinformation as well as blame attribution did not differ based on participants’ age.

By contrast, the Interrogative Suggestibility (IS) framework (Gudjonsson and Clark, 1986) highlighted how individual characteristics can shape a person’s susceptibility to post-event and misleading information during formal questioning. IS is conceived as a dispositional trait and is defined by two primary components: “Yield,” which refers to the tendency of individuals to incorporate details suggested by misleading questions into their responses, and “Shift,” which denotes the propensity to alter answers in response to negative feedback provided by the interviewer during the interrogation process. A critical distinction between other post-event information paradigms (e.g., misinformation) and IS paradigm lies in their sources of misinformation (Dudek and Polczyk, 2024). The post-event information paradigms (e.g., misinformation) focus on independent external sources to provide misleading information, while IS scrutinizes the impact of suggestive questioning within the interrogation context itself. Furthermore, the individual differences approach within the IS framework posits that variability in IS is influenced by personality traits and situational factors that characterize the interrogative environment (Gudjonsson and Clark, 1986). Key factors identified include the degree of uncertainty regarding the correct answer to a question, the level of interpersonal trust in the interviewer’s intentions, and the expectations that interviewees hold regarding the necessity of providing an answer to every inquiry.

One relevant study on IS is the one by Kaasa et al. (2013). In this work, the authors tested whether individual’s suggestibility would make people prone to report false accusations. Specifically, in the first experimental phase, participants interacted with a laboratory assistant (a confederate). During this interaction, the confederate proposed to the participants some personality questionnaires and cognitive tasks and, at the end, measured participants’ height, weight, hip and waist size, skinfold, and blood pressure. In this latter part of the interaction, the confederate had professional and appropriate behavior. However, after this interaction, participants received a telephone call from a second confederate who pretended to be the laboratory supervisor and reported to have received some complaints concerning the assistant’s behavior. Therefore, participants were interviewed twice with leading questions about the interaction with the assistant (i.e., suggesting inappropriate behavior of the assistant). Responses to the leading questions were used to assess individuals’ suggestibility and categorize participants into suggestible and non-suggestible individuals. After the interviews, participants were asked if they wanted to sign a false accusation statement that would result in the assistant being fired. The authors found that suggestible people reported a higher likelihood of signing the complaint concerning the laboratory assistant’s misconduct than people who were not suggestible. These results demonstrated that IS can also have implications on person’s evaluations and behavior.

### External information and evaluative judgments in legal contexts

The above reported brief review showed that a significant body of research has focused on how post-event information can alter the original memory (e.g., Gabbert et al., 2003; Loftus, 2005; Mojtahedi et al., 2017). However, less is known about how such information may influence evaluative judgments about an event. Imagine you are at a park and notice someone sitting on a bench wearing a blue coat. Later, a friend tells you that a suspect, wanted for sexual harassment and described as wearing a black coat, was seen in the same area. When questioned by the police, you report that the person was wearing a black coat, making a memory error. However, what if the police instead asked whether that person seemed suspicious? Could knowing they were suspected of harassment bias your judgment and lead to stronger inferences of guilt?

The influence of external information on evaluation is well-documented in legal contexts, particularly through the lens of pretrial publicity (PTP). Typically, participants are exposed to either prejudicial PTP or neutral information, then review trial materials (e.g., summaries or mock trials) before indicating a verdict or ratings of guilt likelihood. Research shows that exposure to negative PTP about a defendant significantly impacts jurors’ perceptions, shaping their evaluation of trial evidence and leading to biases in verdicts (Ruva and Guenther, 2015; Steblay et al., 1999). A recent metanalysis (Hoetger et al., 2022) summarized and analyzed 45 publications on the impact of PTP on (mock) jurors and jury verdicts. They were interested in understanding how different types of PTP (i.e., negative and positive) may increase or decrease juror vs. jury guilty verdicts and possible moderators (e.g., case type, PTP and trial delay, PTP amount of information, the severity of PTP information) of the effect. They found that negative PTP modestly increases both juror and jury guilty verdict, while positive PTP decreases only juror guilty verdicts. In addition, they found that moderators played a role in these effects. In particular, the effect of both PTP was stronger for specific type of cases (i.e., non-violent crimes), when the delay between the PTP and trial was <1 week, when high amount of PTP information was provided, and when less severe PTP was given to jurors and juries. The typical explanation used to justify PTP effect is the “story model” by Pennington and Hastie’s (1988, 1993). According to this model, jurors build mental narratives that incorporate both trial evidence and external information to make sense of the case. When this narrative is influenced by prejudicial content, it often reinforces guilt-consistent interpretations (Ruva et al., 2007; Ruva and LeVasseur, 2012).

Importantly, even objective-seeming evidence, such as video recordings, can be subject to contextual and framing biases thus influencing evaluative judgments. Ribatti et al. (2024) demonstrated that changes in video speed and narrative wording influenced judgments of intent and accountability in an assault case. For instance, labeling the act as a “hit” versus “harm” or “kill” significantly altered viewers’ attribution of responsibility, with slower videos leading to higher attributions of accountability for the “kill” action. These findings underscore how subtle framing can bias evaluation, particularly when evidence is ambiguous.

Furthermore, biased judgments can arise from internal motivational factors. One of these is the confirmation bias, namely the tendency to seek or interpret information that aligns with their prior beliefs (Nickerson, 1998). This bias is especially dangerous in emotionally charged or evidence-scarce cases like alleged child sexual abuse (Zhang et al., 2022), where interviewers’ expectations can shape not only the questions asked but also the responses elicited, and the conclusions drawn. Such a tendency to interpret ambiguous information in line with prior beliefs extends to jurors as well. In a study by Huntley and Costanzo (2003), mock jurors formed divergent narratives about a sexual harassment case depending on their initial sympathies either portraying the plaintiff as credible or as overly sensitive and complicit. These results highlight how evaluative judgments can reflect not only the facts presented but also the preconceptions and social scripts brought into the interpretive process.

One particularly relevant and perhaps underexplored influence in this context is gender-based schematic processing (Bem, 1981). Cultural stereotypes surrounding gender and power shape how people interpret interpersonal behavior, particularly in ambiguous or emotionally charged situations. For instance, a male authority figure interacting with a younger female subordinate may activate well-established social scripts around misconduct or boundary violations (Eagly and Wood, 2012; Fiske and Taylor, 2013). Arguably, these gendered schemas may increase susceptibility to post-event misinformation, especially when that misinformation aligns with culturally dominant narratives about power and abuse (Rudman and Glick, 2001; Swim et al., 2001). Thus, when ambiguous interactions are later framed in suggestive or accusatory terms, observers may both misremember what occurred and judge the behavior more harshly because that content fits with pre-existing gendered expectations. The social identity theory supports this claim (Turner et al., 1979). This theory explains how people’s self-concept and behavior are influenced by their membership in social groups. It proposes that individuals categorize themselves and others into groups, and that group membership contributes to a sense of identity and self-esteem and, at the same time, contributes to prejudice and stereotypes.

Lindholm and Christianson’s (1998) study provided empirical data of the assumption that gender stereotypes can affect memory along with judgments concerning a person’s behavior. They tested witness’ memory for a violent crime and their evaluations of perpetrator and victim’s behavior. Participants by adopting a witness perspective watched a video depicting a violent crime with a female vs. male perpetrator and a female vs. male victim. They were, then, asked to report their memory for the crime and judge perpetrator’s and victim’s responsibility and culpability for the crime. The authors found that when the gender of the witness (i.e., the participant) and the perpetrator/victim was the same, the witness was more able to report correct details of the perpetrator/victim than when there was no gender congruency between the witness and the perpetrator/victim. Hence, the authors found the same-gender superiority effect in witnesses’ memory when people recalled details of both the perpetrator and victim. In addition, witnesses judged the perpetrator/victim consistently with prevalent gender stereotypes, such that the man perpetrator/victim was perceived as more responsible and culpable than the female.

### The current study

Taken together, the literature depicted above suggests that both memory and judgment might be shaped by a dynamic interplay of pressuring suggestive interview and culturally grounded schemas. In our study, we aimed to investigate these processes by examining how post-event misinformation affects not only memory accuracy for the whole event (through free and cued recall) but also evaluative judgments (i.e., intentionality, appropriateness, severity of the action, level and severity of the punishment), particularly in scenarios involving ambiguous physical touch between individuals in gendered power roles (i.e., professor/student). This is particularly relevant, as legal cases often hinge on witness reports of physical contact, such as in allegations of abuse or sexual harassment. Therefore, examining this issue is crucial for improving the reliability of witness testimony and minimizing bias in legal proceedings. In addition, we were interested in understanding whether the individual’s level of IS would influence the effects of misinformation on memory and evaluative judgments.^1^

Based on the above-mentioned literature, we formulated the following main hypotheses:

*H1*: Individuals who received the misinformation report would provide fewer correct details and more memory errors as compared with participants who did not receive it.

*H2*: Individuals who received the misinformation report would provide higher ratings of intentionality, inappropriateness, severity, level of punishment, and severity of the punishment of professor’s actions as compared with individuals not exposed to misinformation.

*H3*: Individuals who watched the video of the interaction between a male professor and a female student and received the misinformation report would refer fewer correct details and more memory errors, and make higher ratings of intentionality, inappropriateness, severity of the action along with a higher level and severity of the punishment as compared with all other conditions of the design.

*H4*: The individual’s IS would increase the effect of misinformation on memory of correct details, memory errors and evaluative judgments.

## Methods

The study was preregistered on the Open Science Framework (OSF): https://osf.io/43vxc/ (Date created April 22, 2024) and in accordance with the integrity and transparency principles in research, all data and study materials are available on the OSF platform: https://osf.io/43vxc/.

### Participants and design

*A priori* power analysis for a mixed design was run through G*Power (Faul et al., 2007), with *α* = 0.05, power = 0.80, and effect size *f* = 0.25, suggesting a sample size of 180 participants. A total of 184 students (121 women; *M*_age_ = 27.61, *SD* = 10.8) of the Department of Education, Psychology, Communication of the University of Bari Aldo Moro participated in the study. The study was conducted in a laboratory under the supervision of a researcher. Participants did not receive any form of compensation to ensure they were intrinsically motivated to take part in the study.

The experiment adopted a 2*2*2 mixed design with post-event Misinformation (present vs. not present) and actors’ gender Combination (1-male professor/female student vs. 2-female professor/male student) as the between-subjects factors, and Test–retest as the within-subjects factor. The dependent variables were correct details and memory errors (i.e., commissions) reported for free and cued recalls (i.e., misleading and control questions). Additionally, we assessed confidence ratings associated with cued recall items. Finally, we rated intentionality, inappropriateness, severity, level of punishment, and severity of punishment regarding the professor’s actions (i.e., evaluative judgments). Of importance, the index of IS was also included to evaluate the effect of this dispositional trait on participants’ memory and judgments.^2^

The study was approved by the Ethics Committee of the University of Bari Aldo Moro, Department of Education, Psychology Communication (ET-23-20).

### Procedure and measures

The study consisted of two sessions, with a delay of 24 h from each other and lasting approximately 40 and 80 min, respectively. During both sessions participants were tested individually.

#### Session 1

Before starting the study, participants signed an informed consent form and provided socio-demographic information.

##### Video phase

We set up four different short videos, each lasting approximately 2 min. Each participant watched one of these videos displaying an interaction between a university professor (man vs. woman) and a university student (woman vs. man) in the professor’s room. During the interaction (a male professor interacting with a female student or a female professor interacting with a male student), the professor accidentally touches the student either on her/his hand to help her/him moving the mouse (videos 1 and 2), or on his/her shoulder to help her/him taking a book from the bookcase (videos 3 and 4).^3^ Videos are available on OSF: https://osf.io/43vxc/. Participants were randomly assigned to each of the four video conditions.

To approach as much as possible a real-life situation of forensic relevance, participants were asked to imagine themselves as outside observers and pay a particular attention to the details of the interaction (Instructions: “*We are about to show you a video depicting an interaction between a student and a professor during office hours. We kindly ask you to watch the video carefully and imagine that you are witnessing the interaction between student and professor as a person outside the events. That is, we ask you to observe the interaction in the video”*). After watching the video, participants performed a 10-min filler task (i.e., playing the computer game Pinball) to prevent short-term memory effects.

##### Memory test (i.e., free and cued recall)

Participants were first asked to report in detail what they had just seen in the video, trying to be as precise as possible. Then, participants answered 11 cued recall questions related to the video. Specifically, in line with Kaasa et al. (2013)‘s study where the memory questionnaire was composed of questions on actual details of the event and misleading details, we also had 4 misleading questions, namely referring to details not present in the video (e.g., *What color was the professor’s skirt?*”), and 7 control question, namely referring to items seen in the video (e.g., *“How was the Professor dressed?”*). For each of the cued question, we rated participants’ confidence (i.e., *“On a scale of 0 to 10, where 0 is not at all and 10 is completely, how confident are you in the previous answer?”*). Research has shown that post-event information can also influence confidence in their recollection (Semmler et al., 2004; Wright and Skagerberg, 2007), therefore, the confidence rating was included to have a measure of how sure participants were about their recollection. It was proposed also one question concerning the touch (i.e., *“Where on the body did the Professor touch the student?”*). This question was included as a control to check whether people misremember a central detail of the interaction, but it was not included in the main analyses described below (see Note 3).

##### Evaluative judgments

Finally, participants were asked to give their rating on the (a) intentionality, (b) appropriateness, and (c) severity of Professor’s action, and to evaluate (d) the level of punishment the Professor should be given, and (e) the severity of the punishment to be imposed, using a scale ranging from 1 (= not at all) to 10 (= to the highest extent; e.g., *“How intentional do you think the Professor’s touch was?”*).

#### Session 2

##### Misinformation phase

The next day, half of our participants received a misinformation report, and the other half received a neutral report. The misinformation report included information insinuating inappropriate behavior by the Professor (e.g., *“Some students report being touched inappropriately by the Professor”*), whereas the neutral report simply described the Professor’s academic routine without mentioning allegations of misbehavior (e.g., *“The Professor usually reviews students’ examinations”*). Participants who watched the male professor/female student video received the misleading report on the male professor, while those who watched the female professor/male student video during the first session received the misleading report on the female professor.

##### Working memory assessment

Next, the *Wechsler Digit Span Task* (Digit Span; Wechsler, 2008) was performed between the misinformation phase and the final memory test, to (a) assess WM capacity and (b) give participants a filler task. The Digit Span is a measure of verbal short term and WM that can be used in two formats, Forward Digit Span and Reverse Digit Span. Participants are presented with series of digits and are asked to orally reproduce the series either in the order presented (forward span) or in reverse order (backwards span). The test is discontinued if the participant fails two consecutive trials. Total score corresponds to the maximum number of digits the participant was able to correctly repeat.

##### Final memory test (i.e., free and cued recall)

After completing the Digit Span task, participants provided the same free recall and cued recall (i.e., misleading and control questions).

##### Executive functioning and IS assessment

To control for the executive functioning and IS levels, participants were administered the (1) Wisconsin Card Sorting Test (WCST; Heaton et al., 1993), and (2) Gudjonsson Suggestibility Scale 1 (GSS 1; Curci and Bianco, 2014).

The WCST involves 4 stimulus cards and 128 response cards (2 decks of 64 cards), on which figures are represented varying by number (1 to 4 per card), shape (circles, triangles, crosses, or stars), and color (red, blue, yellow, and green). Participants are asked to match each of the response cards with one of the stimulus cards based on a criterion they would find appropriate. They receive feedback from the examiner on the correctness of their match, but they are not told the sorting rule. The participant should use this feedback to identify the correct sorting principle. After correctly sorting 10 cards, the sorting criterion changes without warning, requiring the participant to develop a new classification strategy. Several performance indices are measured during the test. These include (i) the total number of categories completed, (ii) the total number of correct responses, (iii) the number of perseverative errors (sorting according to a previously reinforced category despite negative feedback), (iv) the percentage of non-perseverative errors, and (v) the number of failures to maintain the set.

The GSS 1 is a tool designed to quantify individual susceptibility to leading questions and interrogative pressure. The instrument consists of a narrative paragraph describing a robbery and 20 questions about the story. Specifically, GSS 1 is a verbal memory test where the respondent is asked to recall as much of the story as possible both immediately (immediate recall) and after about 50 min (delayed recall). The interval between these recalls is usually filled with visuo-spatial tasks to avoid verbal interference. In the present study, we filled in the interval with the administration of the WCST. The delayed recall is followed by 20 forced-choice recognition memory items, including 5 yes-no control questions that refer to the actual content of the story and 15 questions about information not included in the story. The answers to these 15 questions provide a measure of the tendency to yield to leading questions (Yield 1 score; range 0–15). After this, the examiner informs the respondent that they made some mistakes, and the same questions are asked again to measure the tendency to change answers under pressure (Yield 2 score; range 0–15). The number of distinct changes in responses following negative feedback is quantified as the Shift score (range 0–20). Total suggestibility is the sum of Yield 1 and Shift and gives an indication of the subject’s overall level of suggestibility (range 0–35). We included this comprehensive IS score in our analyses.

At the end of the experiment, participants were thanked and debriefed about the purpose of the study and answered any questions.

#### Memory scoring

We applied a consistent scoring method across all dependent variables of memory performance (i.e., free recall and cued recall: misleading and control questions). The scoring method is consistent to the one used in prior research (e.g., Battista et al., 2021a; Battista et al., 2021b; Battista et al., 2023). Each response was scored to compute two scores: Correct details score and memory errors score. For free recalls, scores were calculated as follows: The mock crime video was divided into critical units, each referring to an action in the video. To compute the correct details score, 1 point was given to each unit correctly reported (e.g., “*The professor was wearing a blue t-shirt”*), while half a point was assigned for a partially correct unit (e.g., “*The professor was wearing a t-shirt”*). Memory errors score was calculated by attributing 1 point when participants reported a completely wrong detail (e.g., *“The professor was wearing a hat”*) or for every unit not included in the recall, while half a point was assigned when a distorted unit information (e.g., “*The professor was wearing a green t-shirt”*) was referred or when participants partially reported a unit in the recall (e.g., “*The professor was wearing a t-shirt”*). Proportions were calculated by dividing the number of correct details and memory errors reported by the maximum score (i.e., 65 details) (see https://osf.io/43vxc/, for an example).

Similarly, for correct scores of cued recalls, (i) when the response was completely correct, response received 1 point (*“How was the professor dressed?”*; response: *blue t-shirt*), (ii) if it was partially correct, response received 0.5 point (*“How was the professor dressed?”*; response: *t-shirt*), (iii) and if it was completely incorrect, 0 was assigned (*“How was the professor dressed?”*; response: *green jacket*). As to the memory errors scores, (i) when the response was completely correct, response received 0 point (*“How was the professor dressed?”*; response: *blue t-shirt*), (ii) when it was partially wrong or part of the answer was not reported, response received 0.5 point (*“How was the professor dressed?”*; response: *t-shirt*), (iii) and when it was completely incorrect, no response, or “I do not know” was reported, 1 was assigned (*“How was the professor dressed?”*; response: *green jacket*). Proportional scores were then calculated by dividing both the number of correct responses and memory errors by the maximum number of reportable details. For cued recall, this included the sum of misleading questions (e.g., *“What color was the professor’s skirt?”*; maximum of 4) and control questions (e.g., *“How was the professor dressed?”*; maximum of 10).

In our memory test, both at test and retest, we asked participants to respond to a single item referring to the touched area by the professor which was scored dichotomously: 1 point for a correct answer, 0 for an incorrect one. For cued (i.e., misleading and control) questions, we calculated confidence scores. Confidence scores were computed as the rations of the sum of confidences scores for misleading and control questions and the total number of misleading and control questions (respectively, 4 and 7). The scoring system was applied by the first author and FP. The ICC measures for free and cued recall scores at T1 ranged from 0.93 to 1.00 (both *p*s < 0.001) and at T2 from 0.95 to 1.00 (both *p*s < 0.001).

## Results

### Statistical approach

To test the first three hypotheses of our study, a series of 2 (Misinformation: present vs. absent) × 2 (Combination: 1-male professor/female student vs. 2-female professor/male student) × 2 (test–retest) repeated-measures ANOVAs were conducted on (a) correct details and memory errors at the free recall; (b) correct details, memory errors, and confidence levels in response to misleading and control questions; and (c) ratings of intentionality, inappropriateness, severity, level of punishment, and severity of punishment regarding the professor’s actions. The first two factors were treated as between-subjects variables, while test–retest was considered as a within-subjects variable.

Next, Pearson’s correlational analyses were conducted to check the associations between (a) memory scores at the free recall (i.e., correct details and memory errors) and at the cued recall questions (i.e., correct details, memory errors, and confidence), and (b) the evaluative judgments scores (i.e., intentionality, inappropriateness, severity, level of punishment, severity of punishment), for both individual exposed and not exposed to misinformation. Similarly, Pearson correlation analyses were run to evaluate the associations of the GSS Total IS score with correct details and memory error scores for the free recall at test and retest; with correct details, memory errors, and confidence levels in response to cued recall (i.e., misleading and control questions) at test and retest.

Finally, to test H4, namely whether the individual’s disposition to IS influences memory outcomes and evaluative judgments after being exposed to misinformation, a pool of ANCOVAs was conducted with Misinformation (present vs. absent) and Combination (1-male professor/female student vs. 2-female professor/male student) as between subjects factors, Total IS as a covariate, and (a) correct details and memory errors at the free recall task, and at the cued recall (i.e., misleading and control questions) and (b) ratings of intentionality, inappropriateness, severity, level of punishment, and severity of punishment regarding the professor’s actions. All the dependent variables for these ANCOVAs were taken at the retest phase.

We also performed supplementary accuracy-calibration analyses on the correct details at the cued recall (i.e., misleading and control questions). Since we did not have specific a-priori predictions on how confidence would be related to memory accuracy, we added these analyses on OSF: https://osf.io/43vxc/.

### The influence of gender combination and misinformation

In this section, values of significant main and interactions effects are reported, while descriptive values are reported in Table 1.

**Table 1:** Descriptive statistics (i.e., mean and standard deviation) reported at test and retest by combination (Combination 1 vs. Combination 2) and misinformation (present vs. absent) of the memory scores at the free and cued recalls, and judgment ratings.

Measures		Combination 1 (Male professor/Female student)				Combination 2 (Female professor/Male student)			
		Misinformation present		Misinformation absent		Misinformation present		Misinformation absent	
		Test	Retest	Test	Retest	Test	Retest	Test	Retest
Free recall	Correct details	0.30 (0.12)	0.29 (0.09)	0.31 (0.12)	0.27 (0.13)	0.29 (0.09)	0.26 (0.08)	0.26 (0.10)	0.25 (0.10)
	Memory errors	0.04 (0.03)	0.05 (0.04)	0.04 (0.04)	0.03 (0.05)	0.02 (0.02)	0.03 (0.03)	0.02 (0.02)	0.03 (0.03)
Cued recall – Misleading	Correct details	0.77 (0.22)	0.72 (0.25)	0.79 (0.23)	0.77 (0.23)	0.62 (0.23)	0.64 (0.22)	0.68 (0.25)	0.65 (0.22)
	Memory errors	0.21 (0.22)	0.26 (0.25)	0.21 (0.23)	0.22 (0.23)	0.34 (0.21)	0.34 (0.23)	0.31 (0.25)	0.33 (0.22)
	Confidence	9.04 (1.00)	9.14 (1.00)	9.11 (0.83)	9.14 (1.07)	8.86 (1.21)	8.52 (1.26)	8.83 (1.05)	8.76 (0.94)
Cued recall – Control	Correct details	0.52 (0.11)	0.52 (0.13)	0.50 (0.15)	0.50 (0.15)	0.51 (0.11)	0.49 (0.14)	0.52 (0.14)	0.48 (0.14)
	Memory errors	0.29 (0.11)	0.29 (0.13)	0.34 (0.14)	0.33 (0.16)	0.32 (0.11)	0.33 (0.15)	0.29 (0.15)	0.34 (0.16)
	Confidence	8.14 (0.98)	8.15 (1.01)	8.05 (0.95)	8.18 (1.08)	8.09 (1.04)	8.10 (1.07)	7.92 (1.18)	8.04 (1.14)
Judgments	Intentionality	7.96 (2.73)	8.09 (2.56)	7,35 (3.41)	7.28 (4.16)	6.67 (2.97)	6.67 (0.85)	7.00 (2.97)	6.70 (3.05)
	Inappropriateness	7.85 (3.11)	8.24 (2.47)	7.11 (3.20)	6.40 (3.69)	6.20 (3.53)	6.41 (2.91)	6.70 (3.10)	6.20 (3.35)
	Severity	6.74 (3.45)	7.48 (2.87)	6.24 (3.39)	5.70 (3.46)	5.28 (3.54)	5.37 (3.13)	5.22 (3.51)	5.09 (3.55)
	Level of punishment	5.33 (2.99)	6.28 (3.02)	4.15 (3.53)	4.24 (3.43)	3.63 (3.22)	4.35 (3.30)	3.85 (3.51)	4.15 (3.46)
	Severity of punishment	4.83 (2.72)	5.78 (2.81)	4.30 (3.31)	4.02 (3.31)	3.76 (3.20)	4.30 (3.31)	3.72 (3.17)	3.85 (3.39)

#### (a) Free recall

##### (a1) Correct details

**Figure 1:**
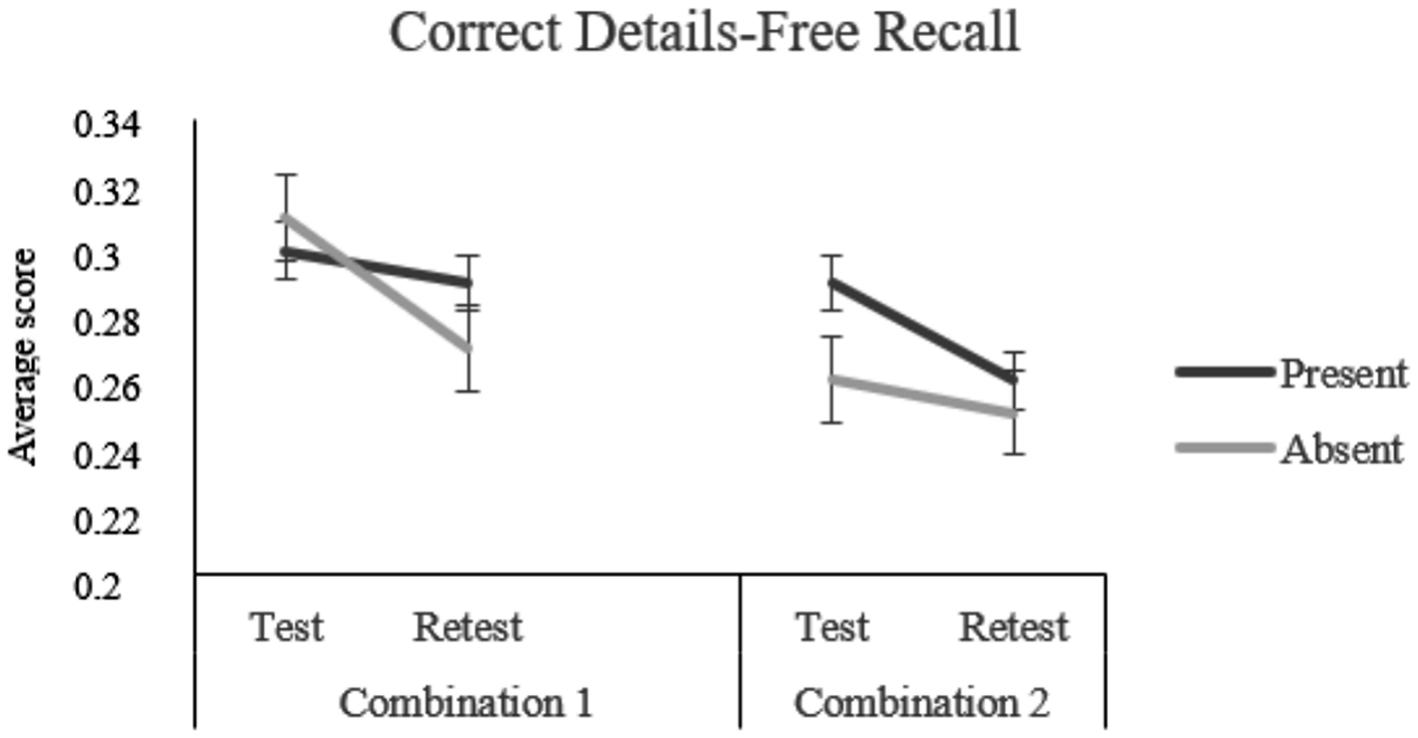
Three-way interaction effects of misinformation (present vs. absent) by combination (1-male professor/female student vs. 2-female professor/male student) by test–retest on the correct details at the free recall.

A significant main effect of Test–retest emerged, *F*(1, 180) = 18.63, *p* < 0.001, *η^2^*_*p*_ = 0.09, indicating less correct details at retest than at test. A significant three-way interaction (Misinformation × Combination × Test–retest) (Figure 1) was found, *F*(1, 180) = 4.46, *p* = 0.036, *η^2^*_*p*_ = 0.024. For participants assigned to Combination 1 and not exposed to misinformation, simple effects analysis confirmed the significant decrease in the amount of reported correct details from test to retest, *F*(1, 180) = 14.42, *p* < 0.001, *n*_*p*_*^2^* = 0.074. On the other hand, for participants assigned to Combination 2 and exposed to misinformation, simple effects analysis confirmed the significant decrease in the amount of reported correct details from test to retest, *F*(1, 180) = 13.27, *p* < 0.001, *n*_*p*_*^2^* = 0.069. No other statistically main nor interaction effects were found significant, *ps* > 0.05.

##### (a2) Memory errors

**Figure 2:**
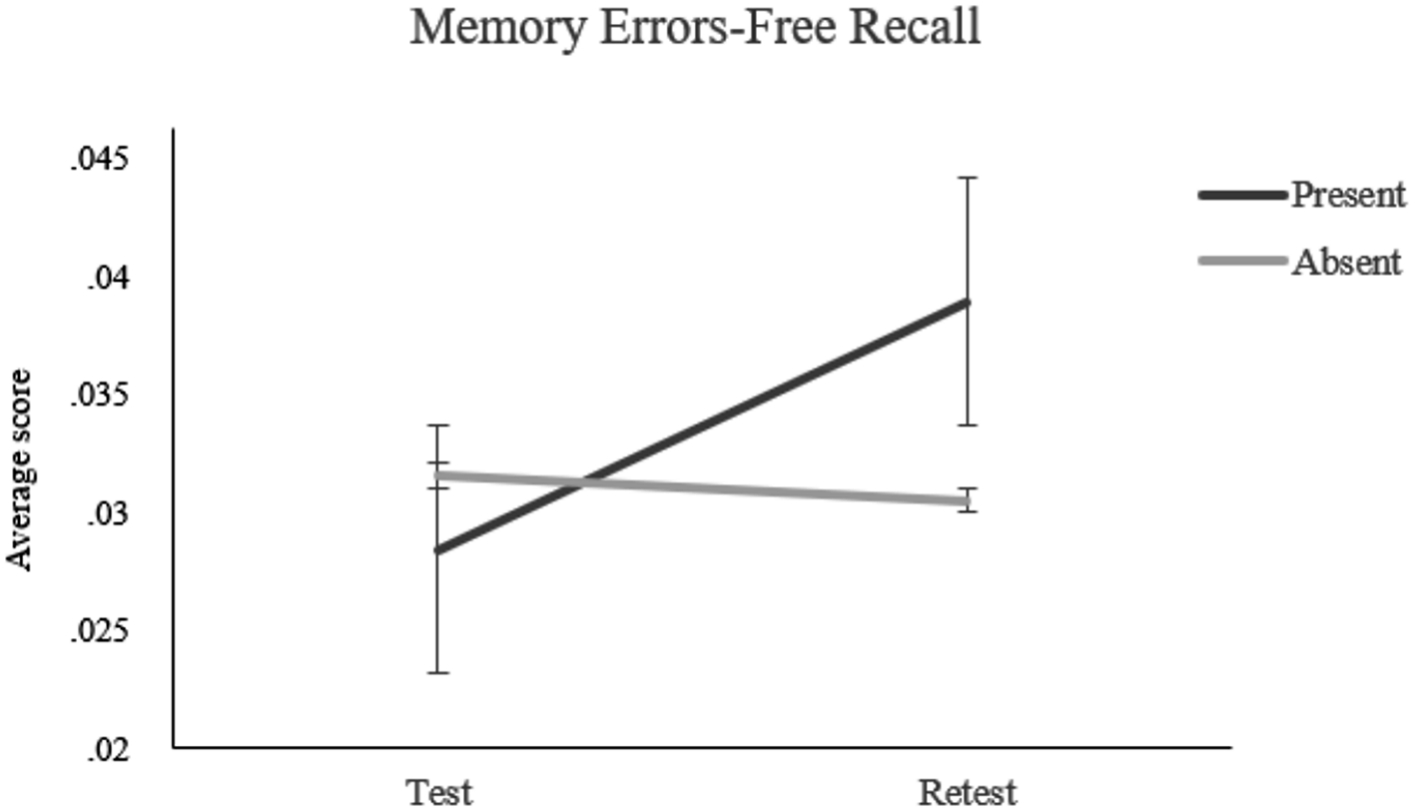
Two-way interaction effect of misinformation (present vs. absent) by test–retest on the memory error scores at the free recall.

A significant main effect of Combination was found, *F*(1, 180) = 13.01, *p* < 0.001, *n*_*p*_*^2^* = 0.067, with more memory errors reported in the Combination 1 condition than in the Combination 2 condition. A significant Misinformation by Test–retest interaction (Figure 2) also emerged, *F*(1, 180) = 5.40, *p* = 0.021, *n*_*p*_*^2^* = 0.029. Simple effects analyses indicated that participants who received misinformation reported more memory errors at retest than test, *F*(1, 180) = 7.76, *p* = 0.007, *n*_*p*_*^2^* = 0.041, with a significant difference from participants not exposed to misinformation at retest, *F*(1, 180) = 4.43, *p* = 0.037, **n*_*p*_ *^2^** = 0.024. No other statistically significant effects were found, *ps* > 0.05.

#### (b1) Cued recall: misleading questions

##### (b1.1) Correct details

A main effect of Combination was observed, *F*(1, 180) = 13.67, *p* < 0.001, **n*_*p*_ *^2^** = 0.071, with more correct details reported in the Combination 1 condition than in the Combination 2 condition. No other statistically significant effects were found, *ps* > 0.05.

##### (b1.2) Memory errors

A significant main effect of Combination emerged, *F*(1, 180) = 11.3, *p* < 0.001, *n*_*p*_*^2^* = 0.060, with more errors observed in the Combination 2 condition than in the Combination 1. No other effects were significant (all *ps* > 0.05).

##### (b1.3) Confidence level

A significant main effect of Combination was found, *F*(1, 180) 7.99, *p* < 0.001, **n*_*p*_ *^2^** = 0.042, indicating higher confidence in the Combination 1 condition compared with the Combination 2 condition. No other effects were statistically significant (all *ps* > 0.05).

#### (b2) Cued recall: control questions

##### (b2.1) Correct details

**Figure 3:**
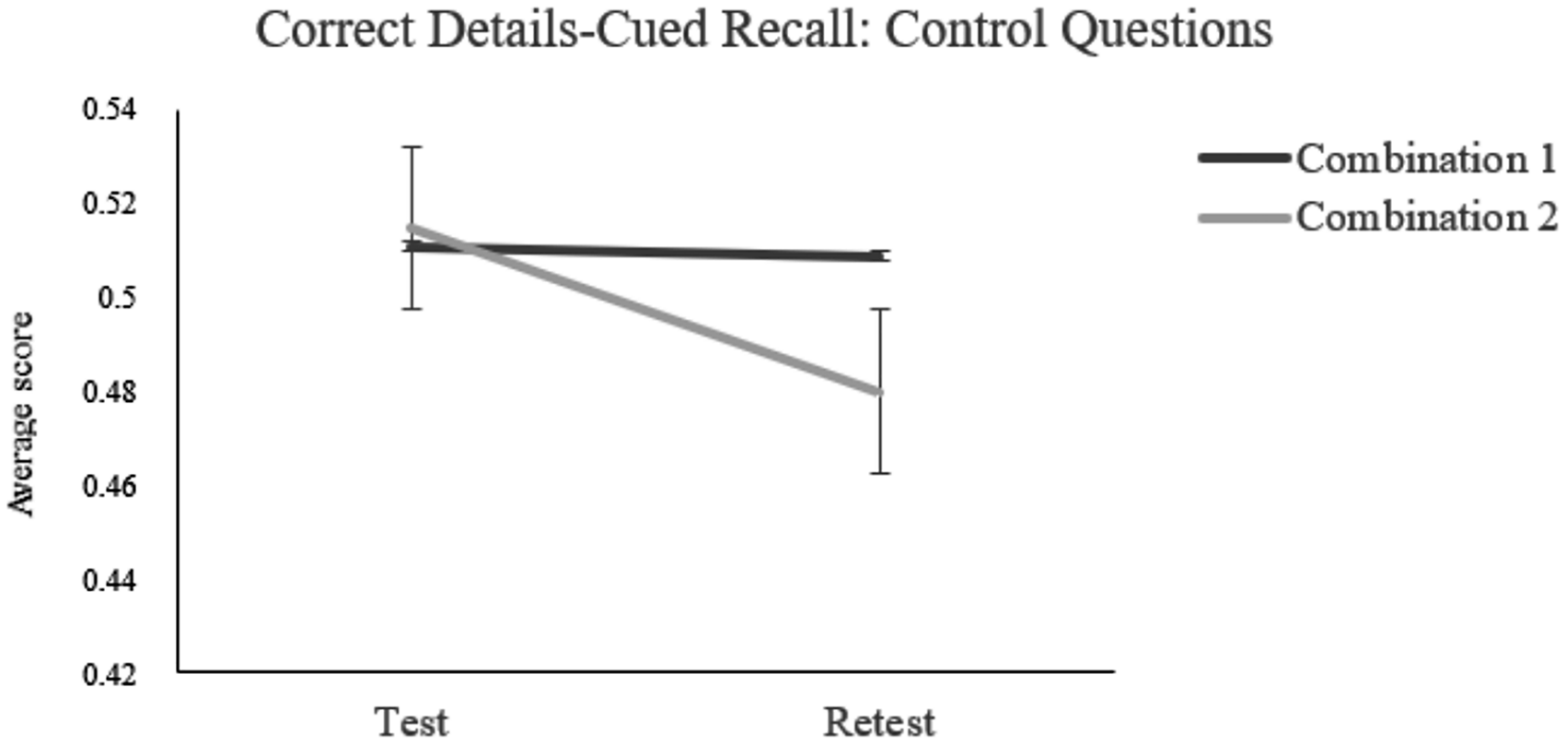
Two-way interaction effect of combination (1-male professor/female student vs. 2-female professor/male student) by test–retest on the correct details scores at the control questions for the cued recall.

A significant main effect of Test–retest was found, *F*(1, 180) = 7.04, *p* = 0.009, *n*_*p*_*^2^* = 0.038, suggesting that the number of correct details in response to control questions was higher at test than retest. The Combination by Test–retest interaction effect (Figure 3) was statistically significant, *F*(1,180) = 5.8, *p* = 0.020, *n*_*p*_*^2^* = 0.030. Simple effects analysis revealed that participants in Combination 2 condition reported a significant decrease in the amount of correct details at retest, *F*(1, 180) = 10.51, *p* = 0.002, *n*_*p*_*^2^* = 0.055. No other effects were statistically significant, all *ps* > 0.05.^4^

##### (b2.2, b2.3) Memory errors and confidence level

No significant effects were found (all *ps* > 0.05).

#### (c) Evaluative judgments

##### (c1) Intentionality

A significant main effect of Combination emerged, *F*(1, 180) = 4.45, *p* = 0.036, *n*_*p*_*^2^* = 0.024, indicating that participants rated the intentionality of the action higher in the Combination 1 condition as compared to the Combination 2 condition. No other significant main effects nor any interaction were found significant (all *ps* > 0.05).

##### (c2) Inappropriateness

**Figure 4:**
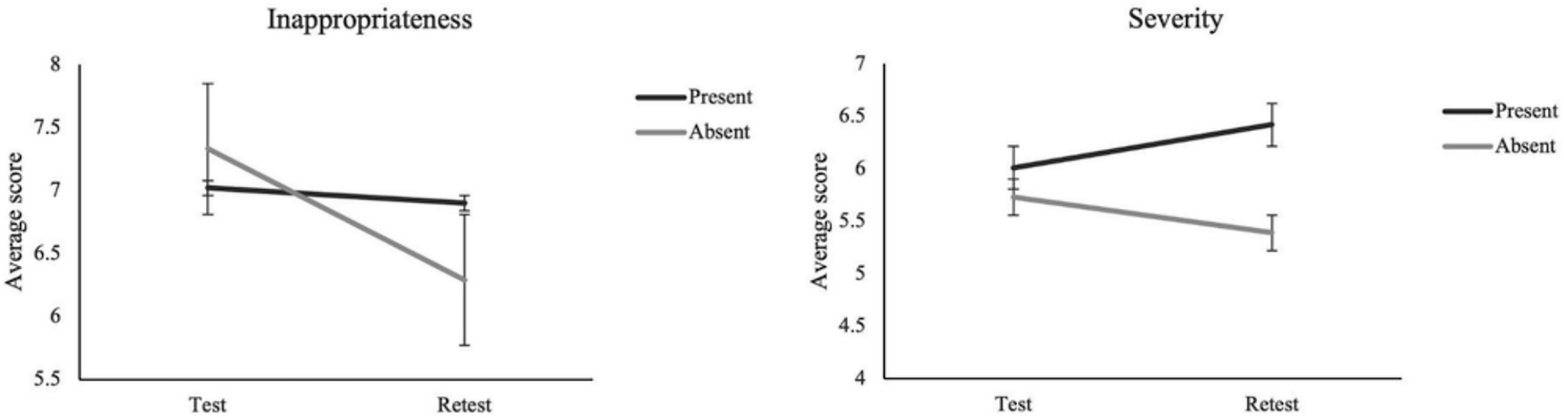
Two-way interaction effects of misinformation (present vs. absent) by test–retest on the inappropriateness and severity scores.

A main effect of Combination was observed, *F*(1, 180) = 4.45, *p* = 0.036, **n*_*p*_ *^2^** = 0.024, with higher ratings of inappropriateness in the Combination 1 condition than in Combination 2. A significant interaction between Misinformation and Test–retest (Figure 4) was observed, *F*(1, 180) = 9.54, *p* = 0.002, **n*_*p*_ *^2^** = 0.050. Simple effects analyses revealed that for participants who did not receive misinformation a significant decrease in the ratings of inappropriateness was observed from test to retest, *F*(1, 180) = 8.69, *p* = 0.004, *n*_*p*_*^2^* = 0.046. In addition, at retest, ratings of inappropriateness were higher for participants exposed to misinformation than for participants not exposed, *F*(1, 180) = 4.98, *p* = 0.027, *n*_*p*_*^2^* = 0.027. No other significant main effects or any interaction effects were found (all *ps* > 0.05).

##### (c3) Severity

A significant main effect of Combination was observed, *F*(1, 180) = 7.44, *p* = 0.007, *n*_*p*_*^2^* = 0.040, with participants in Combination 1 assigning higher severity ratings as compared to participants in Combination 2. A significant Misinformation by Test–retest interaction (Figure 4) was also found, *F*(1, 180) = 7.05, *p* = 0.009, *n*_*p*_*^2^* = 0.038. Simple effects analysis indicated that, at retest, participants exposed to misinformation reported higher severity ratings as compared to participants not exposed to misinformation, *F*(1, 180) = 4.61, *p* = 0.033, *n*_*p*_*^2^* = 0.025. Neither other significant main effects nor any interaction effects were found (all *ps* > 0.05).

##### (c4) Level of punishment

**Figure 5:**
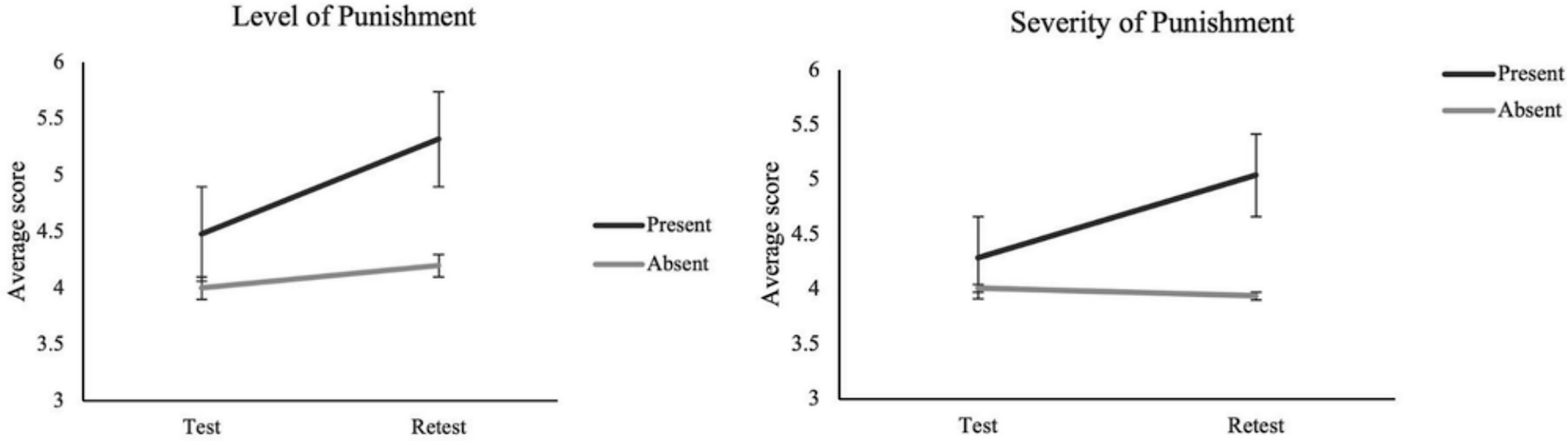
Two-way interaction effects of misinformation (present vs. absent) by test–retest on the level of punishment and severity of punishment scores.

A significant main effect of Test–retest was found, *F*(1, 180) = 16.39, *p* < 0.001, *n*_*p*_*^2^* = 0.083, indicating an overall increase of the ratings at retest as compared to test. A main effect of Combination was also observed, *F*(1, 180) = 4.55, *p* = 0.034, *n*_*p*_*^2^* = 0.025, with a higher level of punishment reported in the Combination 1 condition relative to Combination 2. The Misinformation by test–retest interaction (Figure 5) was significant, *F*(1, 180) = 6.32, *p* = 0.013, *n*_*p*_*^2^* = 0.034. Simple effects analysis revealed that participants exposed to misinformation reported higher scores at retest than at test, *F*(1, 180) = 20.33, *p* < 0.001, *n*_*p*_*^2^* = 0.100. In addition, at retest, participants exposed to misinformation provided significantly higher punishment ratings than participants not exposed to, *F*(1, 180) = 5.27, *p* = 0.023, *n*_*p*_*^2^* = 0.028. No other significant main nor interaction effects were found significant (all *ps* > 0.05).

##### (c5) Severity of the punishment

A main effect of Test–retest was also observed for punishment severity ratings, *F*(1, 180) = 6.44, *p* = 0.012, *n*_*p*_*^2^* = 0.035, showing an overall increase from test to retest. Additionally, the Misinformation by test–retest interaction (Figure 5) was significant, *F*(1, 180) = 9.68, *p* = 0.002, **n*_*p*_ *^2^** = 0.051. Simple effects analyses indicated that at retest participants exposed to misinformation reported higher severity ratings than participants not exposed to, *F*(1, 180) = 5.48, *p* = 0.02, *n*_*p*_*^2^* = 0.029. Moreover, participants exposed to misinformation reported a higher score at retest than at test, *F*(1, 180) = 14.58, *p* < 0.001, *n*_*p*_*^2^* = 0.075. No other significant main nor interaction effects were found to be significant (all *ps* > 0.05).

To summarize, our analyses showed the following results: (i) as to the free recall task, correct details decreased over time, and differences in this decrease was due to misinformation and combination factors; moreover, commissions were higher for participants who watched the male professor/female student interaction than for participants who watched the female professor/male student interaction; (ii) concerning the control questions of the cued recall, correct details decreased over time, especially for participants who watched the female professor/male student interaction; (iii) concerning the misleading questions of the cued recall, correct details were lower and commissions were higher for participants who watched the male professor/female student interaction than those who watched the female professor/male student interaction; (iv) participants exposed to misinformation evaluated the behavior as more serious as compared to the control group, with the exception of intentionality ratings. Additionally, participants who watched the male professor touching the female student provided more severe evaluative judgments than participants watching the female professor touching the male student, except for the perceived severity of punishment for which no effect of the combination factor was observed.

### Associations between memory and judgments

In the group who received misinformation (Table 2), we did not find statistically significant correlations, *p_s_* > 0.05. In the group not exposed to misinformation (Table 3), we only found a positive association between the Inappropriateness score and the Correct Details score at the free recall. No other statistically significant indices were detected, *p_s_* > 0.05.

**Table 2:** Pearson’s correlational indices between memory scores (i.e., free recall, and cued recall) and evaluative judgments at retest in participants who received misinformation.

Evaluative judgments	Free recall		Cued recall			
			Control questions		Misleading questions	
	Correct details	Memory errors	Correct details	Memory errors	Correct details	Memory errors
Intentionality	0.11	0.09	0.05	−0.06	−0.16	0.19
Inappropriateness	0.09	0.03	0.11	−0.17	−0.10	0.13
Severity	0.10	0.03	0.13	−0.19	−0.13	0.15
Level of punishment	0.09	0.04	0.18	−0.10	−0.07	0.08
Severity of punishment	0.02	0.05	0.11	−0.14	−0.15	0.15

*p < 0.05.

**Table 3:** Pearson’s correlational indices between memory scores (i.e., free recall and cued recalls) and evaluative judgments at retest in participants who did not receive misinformation.

Evaluative judgments	Free recall		Cued recall			
			Control questions		Misleading questions	
	Correct details	Memory errors	Correct details	Memory errors	Correct details	Memory errors
Intentionality	0.17	−0.03	0.08	0.01	−0.05	0.05
Inappropriateness	0.25*	0.07	0.15	−0.04	0.17	−0.18
Severity	0.20	0.09	0.07	−0.01	0.08	−0.10
Level of punishment	0.12	0.10	0.05	0.02	0.02	−0.06
Severity of punishment	0.08	0.13	−0.01	0.07	0.02	−0.06

*p < 0.005.

### The role of interrogative suggestibility

No significant correlations were found between GSS Total IS and memory scores at free and cued recalls at test and retest, *p_s_* > 0.05, except for negative correlation between the Total IS and the Correct Detail scores of the free recall at test, *r*(172) = −0.22 *p* = 0.002.^5^

Given that our goal was to verify the role of IS on memory and judgments after the exposure of participants to a misleading report, in the following sections we will report only the effects of the covariate and its interactions with the factors of the design. Overall, we found: (i) no effect of the Total IS score on memory indices (i.e., free recall and cued recall for both misleading and control questions); (ii) regarding evaluative judgments, the Total IS score had a direct effect on intentionality ratings, such that higher IS scores were associated with lower perceived intentionality of the action. In addition, being in the Combination 1 condition maximizes the effect of the Total IS score on the intentionality ratings. No other significant effects of Total IS were observed on judgment measures.

#### (a) Free recall

##### (a1–a2) Correct details and memory errors

The analysis showed neither statistically significant main nor interaction effects, *ps* > 0.05.

##### (b1–b2) Cued recall: misleading and control questions

The analysis reported neither statistically significant main nor interaction effects for correct details, memory errors, and confidence scores, *ps* > 0.05.

#### (c) Evaluative judgments

##### (c1) Intentionality

We found a significant main effect of Total IS, *F*(1, 177) = 11.67, *p* < 0.001, *n*_*p*_*^2^* = 0.062, such that the higher the IS score the lower the ratings of intentionality, *β* = −0.25, *t* = −3.42, *p* < 0.001. Moreover, the interaction of Combination by Total IS score was significant, *F*(1, 177) = 5.12, *p* = 0.025, *η^2^_p_* = 0.029. Specifically, the interaction indicates that being in Combination 1 condition maximized the effect of Total IS on the intentionality judgment, *β* = −0.42, *t* = 3.80, *p* < 0.001. No other significant main nor interactions effects were found to be significant, *ps* > 0.05.

##### (c2) Inappropriateness, (c3) severity, (c4) level of punishment, and (c5) severity of the punishment

The analyses showed neither statistically significant main nor interaction effects for correct details, memory errors, and confidence scores, *ps* > 0.05.

## Discussion

The current study investigated whether exposure to misleading information biased the recall of tactile events (i.e., interaction involving physical touch) and judgments, and how these effects varied depending on the gender composition of the interaction (male professor/female student vs. female professor/male student). Our hypotheses were partially supported by the data, offering contributions to the literature on memory distortion, social cognition, and forensic decision-making.

### The influence of misinformation on memory

Consistent with H1, exposure to post-event misinformation significantly increased memory errors at retest at the free recall for the whole event. This finding replicates the well-established misinformation effect, whereby misleading post-event information impairs the accuracy of memory reports (Chan et al., 2009; Frenda et al., 2011; Loftus, 2005). However, the lack of a direct effect of misleading information on correct recall supports the coexistence hypothesis (Christiaansen and Ochalek, 1983), suggesting that misinformation does not independently overwrite original memories but rather coexists with them in competition during retrieval. This dual-memory representation is also consistent with fuzzy-trace theory (Brainerd and Reyna, 2005), which posits that verbatim and gist traces of memory coexist, with misinformation potentially altering the gist without eliminating the verbatim trace. Moreover, the present results corroborate neurocognitive evidence showing that both true and false memories can activate overlapping but distinct brain regions (Schacter and Slotnick, 2004), further supporting the concept of parallel encoding rather than overwriting. However, our findings also showed that misinformation significantly impairs memory recollection only when participants were exposed to a female professor interacting with a male student, while in the reversed combination condition (male professor vs. female student) misinformation was ineffective. This suggests that the misleading effects may become more influential with delay only under specific characteristics of the target action and its protagonists.

Overall, this study extends prior misinformation research – which has predominantly focused on visual or verbal memory (e.g., Wade et al., 2002). Indeed, our findings suggest that memories related to physical touch are susceptible to distortion when followed by suggestive input, highlighting the malleability of touch-based recall in ambiguous interpersonal contexts.

### Evaluative judgments are biased by misinformation

Consistent with H2, being exposed to misinformation influenced judgments of the professor’s behavior at retest. Specifically, following exposure to misinformation, participants perceived the professor’s actions as inappropriate, severe, and more deserving of punishment, both in terms of level and severity. These misinformation effects emerged only at retest, indicating a delayed impact of misleading information on evaluative judgments. These findings support prior research evidence indicating that memory and moral evaluation are not discrete processes. Rather, these constructs interact dynamically, with memory reconstructions being shaped by post-event beliefs, emotions, and social narratives (Greene and Haidt, 2002).

Our results can be explained also considering research showing a specific form of memory contamination, the blame conformity effect (Thorley and Rushton-Woods, 2013). Studies demonstrating this effect have highlighted that people conform with the co-witness in the attribution of blame for perpetrating a crime because of a memory alteration caused by the exposure to the co-witness report (Mojtahedi et al., 2018a; Mojtahedi et al., 2018b; Thorley, 2015). Our results relate this evidence as we found that, because of the misleading information, participants judged professor’s behavior as more inopportune intense, and punishable.

Moreover, our findings can be explained within the framework of the story model of juror decision-making (Pennington and Hastie, 1993), which posits that individuals interpret evidence by fitting it into coherent narratives, often influenced by framing and prior beliefs. Misinformation may distort the perceived intent behind actions (Winter and Uleman, 1984), particularly when narrative coherence aligns with cultural scripts of wrongdoing. Moreover, people interpret ambiguous or open-to-interpretation evidence in a way that supports their current beliefs, a process known as “double updating,” which can lead to increased polarization even when exposed to the same information as others (Fryer et al., 2019). However, contrary to expectations, misinformation did not increase perceived intentionality. In fact, no significant misinformation effect was found for intentionality judgments, suggesting that participants may have distinguished the professor’s motive from the perceived severity or inappropriateness of the act. Additionally, correlation analyses did not support a direct association between memory accuracy and severity or punishment ratings. This challenges the assumption that better memory necessarily leads to harsher evaluations, and suggests instead that moral judgment may be influenced more by suggestive context and interpretative frames than by the quantity of recalled detail.

### Gender dynamics amplify the effects of misinformation

H3 was not fully supported. While we expected the misinformation effects to be most pronounced in the male professor/female student condition (Combination 1), the results did not show stronger misinformation effects on memory or evaluative judgments in this condition compared to the reverse dyad. Instead, memory performance differed across gender combinations in other unexpected ways. For instance, Combination 1 was associated with more memory errors in free recall but also with better performance on cued recall questions, higher confidence levels, and higher evaluative judgments overall, regardless of misinformation exposure. By contrast, we also found a significant decrease of free recalls due to misinformation for participants watching the female professor touching the male student. These asymmetries may still reflect underlying gender-biased cognitive processing, in which cultural stereotypes and social roles guide interpretations of ambiguous interpersonal behavior (Eagly and Wood, 2012; Fiske and Taylor, 2013).

Overall, our findings converge with Lindholm and Christianson’s (1998) evidence showing that memory and judgments concerning people’s actions- may be shaped by gender stereotypes. Stereotypes influence not only how people view others but also how individuals perceive themselves and their own behaviors, reinforcing gendered expectations in social contexts (Ellemers, 2018). In the present study, the male professor/female student dyad was associated with higher ratings of intentionality, inappropriateness, severity, and punishment, as well as greater confidence in misleading cued recall responses. This pattern suggests that schema-consistent gender dynamics may enhance the subjective clarity and perceived severity of the ambiguous interaction, regardless of memory accuracy. The exposure to male authority figure in a position of power may have activated in our participants pre-existing social schemas associated with misconduct, power imbalance, and sexual harassment, which in turn heightened sensitivity to suggestive information when participants were requested to provide memory free accounts or evaluate the professor’s actions (Rudman and Glick, 2001; Swim et al., 2001).

Moreover, such schema-consistent memory distortions are in line with social identity theory (Turner et al., 1979) and confirmation bias mechanisms (Nickerson, 1998), wherein participants may interpret ambiguous stimuli in ways that confirm culturally dominant narratives (e.g., male authority figures overstepping boundaries with female subordinates). In this study, the male–female dyad likely activated a salient and culturally reinforced script of transgressive touch, enhancing narrative coherence and possibly amplifying subjective certainty. In contrast, participants in the female professor/male student condition (Combination 2) generally showed lower confidence, poorer cued recall, and lower evaluative judgments, possibly reflecting weaker narrative coherence and reduced schema activation. Conversely, in the female professor/male student condition, where such culturally available schemas are weaker or less dominant (Bem, 1981), memory performance was generally poorer, with a cumulative effect of misinformation on free recalls, perhaps likely reflecting reduced narrative coherence or schema incongruence during encoding and retrieval. The same effect of the gender role combination was observed on participants’ confidence in their responses to misleading questions, which has higher in the male professor/female student that in the reverse dyad. Again, this asymmetry may reflect greater schema-consistency where the gendered power dynamic aligns more strongly with culturally available narratives of transgressive behavior (Ellemers, 2018; Rudman and Glick, 2001), potentially fostering a sense of subjective certainty when memory is distorted.

However, despite these asymmetries in confidence and memory consistency, no significant interaction was found between gender combination and misinformation on the overall number of memory errors or evaluative judgments. Therefore, gender dynamics did not seem to amplify the misinformation effect uniformly across all outcome measures.

### Interrogative suggestibility and its effects

We intended to understand whether the level of individual IS could amplify the effects on memory caused by misinformation (H4). Our analysis on the influence of this dispositional trait on memory scores (i.e., free recall scores and misleading and control questions) and evaluative judgments at retest did not completely confirm our hypotheses. Specifically, we only find an association of IS total scores with the scores of free recall at test, but we did not find any effect of IS indices on memory. This means that the individual’s suggestibility trait does not make people more or less likelihood to correctly recall and distort the memory for the original event after being misinformed on that event. An important implication of these findings for the forensic assessment of the eyewitness’ testimony is that the evaluation of the interrogative suggestibility *per se* is insufficient to account for a possible effect of post-event misinformation. Indeed, such an assessment needs to be integrated into a comprehensive consideration of all factors intervening in the experience of providing a witness’ testimony in a police or forensic setting. Parallelling the classical distinction proposed by Wells (1978) and Wells et al. (2020) between estimator and system variables, while IS should be included among the first set of factors impacting upon memory accuracy, a control upon post-event misleading information is compelling to avoid the corruptive effects of the system variables on memory reports.

Finally, IS showed a main effect on participants’ ratings of the professor’s intentionality, irrespective of misinformation exposure. This finding implies that highly suggestible individuals may be more prone to interpreting ambiguous behavior as less intentional, potentially due to a greater reliance on external cues or authority figures for judgment formation. Notably, no associations emerged between suggestibility and ratings of inappropriateness, severity, or punishment. This suggests that while suggestibility may influence perceptions of intent, it does not necessarily generalize to broader moral judgments.

## Limitations and conclusion

Some caveats of the current experiment should be acknowledged. To begin, our participants were mainly students, and this may limit the generalizability of the findings to more diverse or representative populations. Second, while the misinformation was ecologically framed, the laboratory setting may not fully capture the complexity and emotional salience of real-life contexts. Third, the physical touch stimuli were limited to brief and ambiguous interactions, which, although methodologically necessary for control, might not reflect the range of touch scenarios encountered in actual legal cases. Additionally, the reliance on self-reported evaluative ratings may be influenced by individual moral attitudes, which were not directly measured.

Beyond these limits, our findings offer empirical support for the notion that post-event misinformation can systematically distort not only memory for physical-based interactions but also the evaluative judgments individuals make about such actions. Notably, although these distortions were not uniformly moderated by gender characteristics of the protagonists of the action, they were particularly evident in the condition involving a male authority figure, a pattern that likely reflects the activation of culturally shared schemas concerning power, misconduct, and gender roles. These effects underscore the vulnerability of memory and moral judgment to external suggestive input, particularly when ambiguous interpersonal behavior is framed within a suggestive narrative context.

These findings have significant implications for legal procedures, where both factual recall and evaluative impressions frequently inform investigative trajectories and judicial decisions. Professionals operating within the legal system (e.g., investigators, forensic interviewers, judges, and jurors) must remain critically aware of how post-event information may taint witness memory and evaluative reasoning. Hence, particularly in cases involving ambiguous physical touch and socially salient power asymmetries, it is essential to implement procedural safeguards and targeted training to mitigate suggestibility and framing effects during witness evaluation and testimony.

## Data Availability

The datasets presented in this study can be found in online repositories. The names of the repository/repositories and accession number(s) can be found at: https://osf.io/43vxc/.

## Footnotes

1 For exploratory reasons and because of prior evidence showing that individual’s Working Memory and Executive Functions resources can serve as protective factors for false memories formation (Leding, 2012; Mirandola et al., 2017; Peters et al., 2007), we also collected these measures.

2 We also tested participants’ WM and EF abilities as additional individual traits due to studies showing a link between these variables and memory outcomes (e.g., Battista et al., 2020; Battista et al., 2021b; Zhu et al., 2010), however we did not have specific hypotheses for these variables. We included them with exploratory aims.

3 Although participants were exposed to different video versions (i.e., hand touch vs. shoulder touch), we did not include this factor in our main experimental design for two primary reasons. First, the variation in touch location was introduced to preserve ecological validity and introduce natural variability in the professors’ actions. Second, we had no *a priori* hypotheses regarding whether the specific body part touched would influence memory and/or evaluative judgments (to our knowledge, there is nothing specific on this matter in the existing literature). However, for exploratory purposes, we included the variable “touch” (hand vs. shoulder) in the repeated measures ANOVA models described in the subsequent sections and we observed no relevant changes in the pattern of results obtained. A complete report of these analyses is available in Supplementary Material (https://osf.io/43vxc/).

4 As specified in the section on Measures, the item concerning the touched area by the professor was primarily intended as a control for misremembering a central detail of the interaction, so that it was not considered for the subsequent analyses. However, explored the (mis)remembering of this detail across the cells of the design at both test and retest. Overall, the part of the student’s body that was touched (hand or shoulder) was correctly reported by 83.5% of the participants at test and by 81% of the participants at retest, with no significant differences for misinformation and combination factors. Importantly, participants who did not report the correct body part touched typically mentioned a body part close to the correct one (e.g., forearm, arm), at both test and retest. Additional details are available in Supplementary material (https://osf.io/43vxc/).

5 Pearson correlation analyses were also conducted to evaluate the association of Digit Span scores with (a) correct details and memory errors in the free recall; (b) correct details, memory errors, and confidence levels in response to misleading and control questions at test and retest. No significant correlations were found, *p*s > 0.05. Similar correlation analyses were carried out on the Wisconsin Card Sorting Test scores of total correct responses, and total errors. No significant correlations were found, *p*s > 0.05.

## References

[ref1] Alba J. W. Hasher L. (1983). Is memory schematic? Psychol. Bull. 93, 203–231. doi: 10.1037/0033-2909.93.2.203

[ref2] Arnold M. M. Lindsay D. S. (2002). Remembering remembering. J. Exp. Psychol. Learn. Mem. Cogn. 28, 521–529. doi: 10.1037/0278-7393.28.3.521, 12018504

[ref3] Battista F. Lanciano T. Curci A. (2021a). Does alexithymia affect memory for a crime? The relationship between alexithymia, executive functions, and memories. Front. Psychol. 12:669778. doi: 10.3389/fpsyg.2021.669778, 34276491 PMC8278017

[ref4] Battista F. Lanciano T. Curci A. Mirandola C. Otgaar H. (2023). I lie because I am good at: psychopathic traits do not influence the effects of fabrication on memory. J. Cogn. Psychol. 35, 635–649. doi: 10.1080/20445911.2023.2245603

[ref5] Battista F. Mangiulli I. Otgaar H. Curci A. (2024). The impact of internal and external influences on memory and their relevance to legal decisions. Front. Psychol. 15:1408797. doi: 10.3389/fpsyg.2024.1408797, 38868354 PMC11167071

[ref6] Battista F. Otgaar H. Lanciano T. Curci A. (2020). Individual differences impact memory for a crime: a study on executive functions resources. Conscious. Cogn. 84:103000. doi: 10.1016/j.concog.2020.103000, 32828004

[ref7] Battista F. Otgaar H. Mangiulli I. Curci A. (2021b). The role of executive functions in the effects of lying on memory. Acta Psychol. 215:103295. doi: 10.1016/j.actpsy.2021.103295 33752141

[ref8] Bem S. L. (1981). Gender schema theory: a cognitive account of sex typing. Psychol. Rev. 88, 354–364. doi: 10.1037/0033-295X.88.4.354

[ref9] Brainerd C. J. Reyna V. F. (1998). Fuzzy-trace theory and children's false memories. J. Exp. Child Psychol. 71, 81–129. doi: 10.1006/jecp.1998.2464, 9843617

[ref10] Brainerd C. J. Reyna V. F. Ceci S. J. (2008). Developmental reversals in false memory: a review of data and theory. Psychol. Bull. 134, 343–382. doi: 10.1037/0033-2909.134.3.343, 18444700

[ref001] Brainerd C. J. Reyna V. F. (2005). The science of false memory. Oxford University Press.,

[ref11] Bransford J. D. Johnson M. K. (1972). Contextual prerequisites for understanding: some investigations of comprehension and recall. J. Verbal Learn. Verbal Behav. 11, 717–726. doi: 10.1016/S0022-5371(72)80006-9

[ref12] Brassil M. O’Mahony C. Greene C. M. (2024). Do cognitive abilities reduce eyewitness susceptibility to the misinformation effect? A systematic review. Psychon. Bull. Rev. 31, 2410–2436. doi: 10.3758/s13423-024-02512-5, 38696106 PMC11680610

[ref13] Chan J. C. Thomas A. K. Bulevich J. B. (2009). Recalling a witnessed event increases eyewitness suggestibility: the reversed testing effect. Psychol. Sci. 20, 66–73. doi: 10.1111/j.1467-9280.2008.02245.x, 19037905

[ref14] Christiaansen R. E. Ochalek K. (1983). Editing misleading information from memory: evidence for the coexistence of original and postevent information. Mem. Cogn. 11, 467–475. doi: 10.3758/BF03196983, 6656606

[ref15] Curci A. (2022). Autobiographical memory and constructive processes: from flashbulb memories to eyewitness’ memory. G. Ital. Psicol. 49, 197–202. doi: 10.1421/104965

[ref16] Curci A. Bianco A. (2014). Gudjonsson suggestibility scales. Firenze: Manuale d’uso Giunti O.S. Organizzazioni Speciali.

[ref17] Dudek I. Polczyk R. (2024). Memory distrust and suggestibility: a registered report. Leg. Criminol. Psychol. 29, 100–123. doi: 10.1111/lcrp.12297

[ref18] Eagly A. H. Wood W. (2012). Social role theory. In: Lange P. A. M. Van Kruglanski A. W. Higgins E. T. (Eds.), Handbook of theories of social psychology. Vol. 2. Thousand Oaks, CA: Sage Publications Ltd.; 458–476

[ref19] Ellemers N. (2018). Gender stereotypes. Annu. Rev. Psychol. 69, 275–298. doi: 10.1146/annurev-psych-122216-011719, 28961059

[ref20] Faul F. Erdfelder E. Lang A. G. Buchner A. (2007). G* power 3: a flexible statistical power analysis program for the social, behavioral, and biomedical sciences. Behav. Res. Methods 39, 175–191. doi: 10.3758/BF03193146, 17695343

[ref21] Fiske S. T. T. Taylor S. E. (2013). Social Cognition: From Brains to Culture: Sage Publication. doi: 10.4135/9781529681451

[ref22] Frenda S. J. Nichols R. M. Loftus E. F. (2011). Current issues and advances in misinformation research. Curr. Dir. Psychol. Sci. 20, 20–23. doi: 10.1177/0963721410396620

[ref23] Fryer R. G. Jr. Harms P. Jackson M. O. (2019). Updating beliefs when evidence is open to interpretation: implications for bias and polarization. J. Eur. Econ. Assoc. 17, 1470–1501. doi: 10.1093/jeea/jvy025

[ref24] Gabbert F. Memon A. Allan K. (2003). Memory conformity: can eyewitnesses influence each other's memories for an event? Appl. Cogn. Psychol. 17, 533–543. doi: 10.1002/acp.885

[ref26] Greene J. Haidt J. (2002). How (and where) does moral judgment work? Trends Cogn. Sci. 6, 517–523. doi: 10.1016/S1364-6613(02)02011-9, 12475712

[ref27] Gudjonsson G. H. Clark N. K. (1986). Suggestibility in police interrogation: a social psychological model. Soc. Behav. 1, 83–104.

[ref28] Heaton R. K. Chelune G. J. Talley J. L. Kay G. G. Curtiss G. (1993). Wisconsin card sorting test manual: Revised and expanded: Psychological Assessment Resources.

[ref29] Hoetger L. A. Devine D. J. Brank E. M. Drew R. M. Rees R. (2022). The impact of pretrial publicity on mock juror and jury verdicts: a meta-analysis. Law Hum. Behav. 46, 121–139. doi: 10.1037/lhb0000473, 35084906

[ref30] Huntley J. E. Costanzo M. (2003). Sexual harassment stories: testing a story-mediated model of juror decision-making in civil litigation. Law Hum. Behav. 27, 29–51. doi: 10.1023/A:1021685519379, 12647466

[ref31] Johnson M. K. Hashtroudi S. Lindsay D. S. (1993). Source monitoring. Psychol. Bull. 114, 3–28. doi: 10.1037/0033-2909.114.1.3, 8346328

[ref32] Kaasa S. O. Cauffman E. Alison Clarke-Stewart K. Loftus E. F. (2013). False accusations in an investigative context: differences between suggestible and non-suggestible witnesses. Behav. Sci. Law 31, 574–592. doi: 10.1002/bsl.2075, 23852883

[ref33] Kassin S. M. Goldstein C. C. Savitsky K. (2003). Behavioral confirmation in the interrogation room: on the dangers of presuming guilt. Law Hum. Behav. 27, 187–203. doi: 10.1023/A:1022599230598, 12733421

[ref34] Leding J. K. (2012). Working memory predicts the rejection of false memories. Memory 20, 217–223. doi: 10.1080/09658211.2011.653373, 22292532

[ref35] Lindholm T. Christianson S. Å. (1998). Gender effects in eyewitness accounts of a violent crime. Psychol. Crime Law 4, 323–339. doi: 10.1080/10683169808401763

[ref36] Loftus E. F. (1975). Leading questions and the eyewitness report. Cogn. Psychol. 7, 560–572. doi: 10.1016/0010-0285(75)90023-7

[ref37] Loftus E. F. (1997). Creating false memories. Sci. Am. 277, 70–75. doi: 10.1038/scientificamerican0997-70, 9274041

[ref38] Loftus E. F. (2005). Planting misinformation in the human mind: a 30-year investigation of the malleability of memory. Learn. Mem. 12, 361–366. doi: 10.1101/lm.94705, 16027179

[ref39] Loftus E. F. Miller D. G. Burns H. J. (1978). Semantic integration of verbal information into a visual memory. J. Exp. Psychol. Hum. Learn. Mem. 4, 19–31. doi: 10.1037/0278-7393.4.1.19, 621467

[ref40] Loftus E. F. Pickrell J. E. (1995). The formation of false memories. Psychiatr. Ann. 25, 720–725. doi: 10.3928/0048-5713-19951201-07

[ref41] Mazzoni G. A. Vannucci M. Loftus E. F. (1999). Misremembering story material: the role of causal connections. Leg. Criminol. Psychol. 4, 93–110. doi: 10.1348/135532599167728

[ref42] Melinder A. Brennen T. Husby M. F. Vassend O. (2020). Personality, confirmation bias, and forensic interviewing performance. Appl. Cogn. Psychol. 34, 961–971. doi: 10.1002/acp.3674

[ref43] Mirandola C. Toffalini E. Ciriello A. Cornoldi C. (2017). Working memory affects false memory production for emotional events. Cognit. Emot. 31, 33–46. doi: 10.1080/02699931.2015.1075379, 26316214

[ref44] Mitchell K. J. Johnson M. K. (2000). “Source monitoring: attributing mental experiences” in The Oxford handbook of memory. eds. Tulving E. Craik F. I. M. (New York: Oxford University Press), 179–195.

[ref45] Mojtahedi D. Ioannou M. Hammond L. (2017). Personality correlates of co-witness suggestibility. J. Forensic Psychol. Res. Pract. 17, 249–274. doi: 10.1080/24732850.2017.1358996

[ref46] Mojtahedi D. Ioannou M. Hammond L. (2018a). Group size, misinformation and unanimity influences on co-witness judgements. J. Forensic Psychiatry Psychol. 29, 844–865. doi: 10.1080/14789949.2018.1439990

[ref47] Mojtahedi D. Ioannou M. Hammond L. (2018b). The dangers of co-witness familiarity: investigating the effects of co-witness relationships on blame conformity. J. Police Crim. Psychol. 33, 316–326. doi: 10.1007/s11896-018-9254-4

[ref48] Mojtahedi D. Ioannou M. Hammond L. (2020). Intelligence, authority and blame conformity: co-witness influence is moderated by the perceived competence of the information source. J. Police Crim. Psychol. 35, 422–431. doi: 10.1007/s11896-019-09361-2

[ref49] Nash R. A. Wheeler R. L. Hope L. (2015). On the persuadability of memory: is changing people's memories no more than changing their minds? Br. J. Psychol. 106, 308–326. doi: 10.1111/bjop.12085, 24898340

[ref50] Nickerson R. S. (1998). Confirmation bias: a ubiquitous phenomenon in many guises. Rev. Gen. Psychol. 2, 175–220. doi: 10.1037/1089-2680.2.2.175

[ref51] Otgaar H. Houben S. T. Howe M. L. (2018). “Methods of studying false memory” in Handbook of research methods in human memory (New York: Routledge), 238–252.

[ref52] Otgaar H. Mangiulli I. Battista F. Howe M. L. (2023). External and internal influences yield similar memory effects: the role of deception and suggestion. Front. Psychol. 14:1081528. doi: 10.3389/fpsyg.2023.1081528, 37701866 PMC10494980

[ref54] Pennington N. Hastie R. (1988). Explanation-based decision making: effects of memory structure on judgment. J. Exp. Psychol. Learn. Mem. Cogn. 14, 521–533. doi: 10.1037/0278-7393.14.3.521

[ref55] Pennington N. Hastie R. (1993). “The story model for juror decision making” in Inside the juror: The psychology of juror decision making. ed. Hastie R. (Cambridge University Press), 192–221.

[ref56] Peters M. J. Jelicic M. Verbeek H. Merckelbach H. (2007). Poor working memory predicts false memories. Eur. J. Cogn. Psychol. 19, 213–232. doi: 10.1080/09541440600760396

[ref57] Pickrell J. E. McDonald D. L. Bernstein D. M. Loftus E. F. (2016). “Misinformation effect” in Cognitive illusions. ed. Pohl R. (Psychology Press), 406–423.

[ref58] Reyna V. F. Corbin J. C. Weldon R. B. Brainerd C. J. (2016). How fuzzy-trace theory predicts true and false memories for words, sentences, and narratives. J. Appl. Res. Mem. Cogn. 5, 1–9. doi: 10.1016/j.jarmac.2015.12.003, 27042402 PMC4815269

[ref59] Ribatti R. M. Lanciano T. De’Sperati C. Curci A. (2024). Speed and contextual information of a crime-related video bias the responsibility judgments. Curr. Psychol. 43, 25403–25413. doi: 10.1007/s12144-023-05385-3

[ref60] Roediger H. L. McDermott K. B. (1995). Creating false memories: remembering words not presented in lists. J. Exp. Psychol. Learn. Mem. Cogn. 21, 803–814. doi: 10.1037/0278-7393.21.4.803, 41340023

[ref61] Roediger H. L. McDermott K. B. Robinson K. J. (2014). “The role of associative processes in creating false memories” in Theories of memory II. eds. Gazzaniga M. S. Mangun G. R., (Taylor and Francis). 187–245.

[ref62] Rudman L. A. Glick P. (2001). Prescriptive gender stereotypes and backlash toward agentic women. J. Soc. Issues 57, 743–762. doi: 10.1111/0022-4537.00239

[ref63] Ruva C. L. Guenther C. C. (2015). From the shadows into the light: how pretrial publicity and deliberation affect mock jurors’ decisions, impressions, and memory. Law Hum. Behav. 39, 294–306. doi: 10.1037/lhb0000136, 25495716

[ref64] Ruva C. L. LeVasseur M. A. (2012). Behind closed doors: the effect of pretrial publicity on jury deliberations. Psychol. Crime Law 18, 431–452. doi: 10.1080/1068316X.2010.505567

[ref65] Ruva C. McEvoy C. Bryant J. B. (2007). Effects of pre-trial publicity and jury deliberation on juror bias and source memory errors. Appl. Cogn. Psychol. 21, 45–67. doi: 10.1002/acp.1265

[ref66] Schacter D. L. (1999). The seven sins of memory: insights from psychology and cognitive neuroscience. Am. Psychol. 54, 182–203. doi: 10.1037/0003-066X.54.3.182, 10199218

[ref67] Schacter D. L. Addis D. R. Buckner R. L. (2007). Remembering the past to imagine the future: the prospective brain. Nat. Rev. Neurosci. 8, 657–661. doi: 10.1038/nrn2213, 17700624

[ref68] Schacter D. L. Slotnick S. D. (2004). The cognitive neuroscience of memory distortion. Neuron 44, 149–160. doi: 10.1016/j.neuron.2004.08.017, 15450167

[ref69] Semmler C. Brewer N. Wells G. L. (2004). Effects of postidentification feedback on eyewitness identification and nonidentification confidence. J. Appl. Psychol. 89, 334–346. doi: 10.1037/0021-9010.89.2.334, 15065979

[ref70] Steblay N. M. Besirevic J. Fulero S. M. Jimenez-Lorente B. (1999). The effects of pretrial publicity on juror verdicts: a meta-analytic review. Law Hum. Behav. 23, 219–235. doi: 10.1023/A:1022325015110

[ref71] Swim J. K. Hyers L. L. Cohen L. L. Ferguson M. J. (2001). Everyday sexism: evidence for its incidence, nature, and psychological impact from three daily diary studies. J. Soc. Issues 57, 31–53. doi: 10.1111/0022-4537.00200

[ref72] Thorley C. (2015). Blame conformity: innocent bystanders can be blamed for a crime as a result of misinformation from a young, but not elderly, adult co-witness. PLoS One 10:e0134739. doi: 10.1371/journal.pone.0134739, 26230523 PMC4521947

[ref73] Thorley C. Rushton-Woods J. (2013). Blame conformity: leading eyewitness statements can influence attributions of blame for an accident. Appl. Cogn. Psychol. 27, 291–296. doi: 10.1002/acp.2906

[ref74] Tousignant J. P. Hall D. Loftus E. F. (1986). Discrepancy detection and vulnerability to misleading postevent information. Mem. Cogn. 14, 329–338. doi: 10.3758/BF03202511, 3762387

[ref75] Turner J. C. Brown R. J. Tajfel H. (1979). Social comparison and group interest in ingroup favouritism. Eur. J. Soc. Psychol. 9, 187–204. doi: 10.1002/ejsp.2420090207

[ref76] Wade K. A. Garry M. Don Read J. Lindsay D. S. (2002). A picture is worth a thousand lies: using false photographs to create false childhood memories. Psychon. Bull. Rev. 9, 597–603. doi: 10.3758/BF03196318, 12412902

[ref77] Wechsler D. (2008). Wechsler adult intelligence scale. 4th Edn: Pearson.

[ref78] Wells G. L. (1978). Applied eyewitness-testimony research: system variables and estimator variables. J. Pers. Soc. Psychol. 36, 1546–1557. doi: 10.1037/0022-3514.36.12.1546

[ref79] Wells G. L. Memon A. Penrod S. D. (2020). Eyewitness evidence: improving its probative value. Psychol. Sci. Public Interest 7, 45–75. doi: 10.1111/j.1529-1006.2006.00027.x, 26158855

[ref81] Winter L. Uleman J. S. (1984). When are social judgments made? Evidence for the spontaneousness of trait inferences. J. Pers. Soc. Psychol. 47, 237–252. doi: 10.1037/0022-3514.47.2.237, 6481615

[ref82] Wright D. B. Self G. Justice C. (2000). Memory conformity: exploring misinformation effects when presented by another person. Br. J. Psychol. 91, 189–202. doi: 10.1348/000712600161781, 10832514

[ref83] Wright D. B. Skagerberg E. M. (2007). Postidentification feedback affects real eyewitnesses. Psychol. Sci. 18, 172–178. doi: 10.1111/j.1467-9280.2007.01868, 17425539

[ref84] Zhang Y. Segal A. Pompedda F. Haginoya S. Santtila P. (2022). Confirmation bias in simulated CSA interviews: how abuse assumption influences interviewing and decision-making processes? Leg. Criminol. Psychol. 27, 314–328. doi: 10.1111/lcrp.12216

[ref85] Zhu B. Chen C. Loftus E. F. Lin C. He Q. Chen C. . (2010). Individual differences in false memory from misinformation: cognitive factors. Memory 18, 543–555. doi: 10.1080/09658211.2010.487051, 20623420

